# Supplementary material for: Integrative frontal-parietal dynamics supporting cognitive control
Source: eLife. 2021 Mar 2;10:e57244. doi: 10.7554/eLife.57244 (PMC7963482; doi:10.7554/eLife.57244)
Supplement: Supplementary file 1. — Factorial ANOVA’s for the sub-task trials and return trials. RT – reaction time; ACC – accuracy; ME – main effect. [file elife-57244-supp1.docx]

**Supplemental Table 1 – Sub-Task Behavioral Data**

|  | Samp 1 |  |  |  | Samp 2 |  |  |  | Combine |  |  |  |
| --- | --- | --- | --- | --- | --- | --- | --- | --- | --- | --- | --- | --- |
|  | RT |  | ACC |  | RT |  | ACC |  | RT |  | ACC |  |
| Effect | F(1,23) | p-value | F(1,23) | p-value | F(1,24) | p-value | F(1,24) | p-value | F(1,48) | p-value | F(1,48) | p-value |
| ME Stim Domain | 0.03 | 0.8691 | 3.12 | 0.0905 | **5.01** | **0.0348** | 2.90 | 0.1015 | 3.55 | 0.0657 | **6.09** | **0.0172** |
| ME Context Control | **150.96** | **<0.0001** | **16.22** | **0.0005** | **144.39** | **<0.0001** | **42.69** | **<0.0001** | **198.37** | **<0.0001** | **53.37** | **<0.0001** |
| ME Temp Control | **24.23** | **0.0001** | **5.71** | **0.0255** | **11.22** | **0.0027** | **5.45** | **0.0283** | **26.50** | **<0.0001** | **11.23** | **0.0016** |
| Stim Domain X Context Control | 0.04 | 0.8395 | 0.04 | 0.8392 | 0.14 | 0.7091 | 0.38 | 0.5414 | 0.05 | 0.831 | 0.06 | 0.8019 |
| Stim Domain X Temp Control | **7.09** | **0.0139** | 0.13 | 0.7172 | 1.82 | 0.1900 | 0.33 | 0.5723 | **5.96** | **0.0184** | 0.06 | 0.8101 |
| Context Control x Temp Control | **96.13** | **<0.0001** | **23.79** | **0.0001** | **105.04** | **<0.0001** | **7.44** | **0.0118** | **204.98** | **<0.0001** | **27.12** | **<0.0001** |
| Stim Domain x Context Control x Temp Control | 0.03 | 0.8559 | 0.24 | 0.6321 | <0.01 | 0.9729 | 0.49 | 0.4892 | <0.01 | 0.9467 | 0.01 | 0.9412 |

**Supplemental Table 2 – Return Behavioral Data**

|  | Samp 1 |  |  |  | Samp 2 |  |  |  | Combine |  |  |  |
| --- | --- | --- | --- | --- | --- | --- | --- | --- | --- | --- | --- | --- |
|  | RT |  | ACC |  | RT |  | ACC |  | RT |  | ACC |  |
| Effect | F(1,23) | p-value | F(1,23) | p-value | F(1,24) | p-value | F(1,24) | p-value | F(1,48) | p-value | F(1,48) | p-value |
| ME Stim Domain | 0.12 | 0.7369 | 3.01 | 0.0961 | 3.80 | 0.063 | 0.01 | 0.9057 | 2.32 | 0.1345 | 1.62 | 0.2091 |
| ME Context Control | **85.74** | **<0.0001** | **20.00** | **0.0002** | **145.46** | **<0.0001** | **38.88** | **<0.0001** | **216.36** | **<0.0001** | **57.08** | **<0.0001** |
| ME Temp Control | 3.79 | 0.064 | **11.44** | **0.0026** | 3.09 | 0.0913 | 3.79 | 0.0634 | **6.9** | **0.0116** | **14.43** | **0.0004** |
| Stim Domain X Context Control | 0.74 | 0.3986 | 3.95 | 0.0588 | 0.35 | 0.5576 | 0.49 | 0.4907 | 0.96 | 0.3329 | 2.79 | 0.1012 |
| Stim Domain X Temp Control | 1.02 | 0.3223 | 0.12 | 0.7326 | 0.39 | 0.5357 | 1.18 | 0.2352 | 0.01 | 0.937 | 0.64 | 0.4269 |
| Context Control x Temp Control | **8.66** | **0.0073** | **19.19** | **0.0002** | **6.77** | **0.0156** | 1.66 | 0.2103 | **15.45** | **0.0003** | **10.22** | **0.0025** |
| Stim Domain x Context Control x Temp Control | 1.48 | 0.2364 | 1.99 | 0.172 | 0.39 | 0.5384 | 0.01 | 0.9306 | 1.68 | 0.2015 | 0.32 | 0.5761 |
